# The challenges of becoming and being a clinician manager: a qualitative exploration of the perception of medical doctors in senior leadership roles at a large Australian health service

**DOI:** 10.1186/s12913-021-06356-w

**Published:** 2021-04-15

**Authors:** Didir Imran, Karen Rog, John Gallichio, Laura Alston

**Affiliations:** 1 Colac Area Health, Colac Victoria, Australia; 2 Western Health, Melbourne, Victoria Australia; 3 Global Obesity Centre, Institute for Health Transformation, Deakin University, Deakin, Victoria Australia; 4 Deakin Rural Health, Faculty of Health, Deakin University, Deakin, Victoria Australia

**Keywords:** Doctors, Leadership, Clinician managers, Challenges

## Abstract

**Background:**

In Australia, activity-based funding models have emphasized the need for hospitals to be accountable for their clinical performance. Clinician managers, with medical backgrounds are essential to ensuring high quality clinical performance and operational management of hospital services. The purpose of this study is to 1. Identify factors influencing doctors to become clinician managers in the Australian healthcare setting. 2. Understand the pathways and challenges faced by doctors in becoming clinician managers.

**Methods:**

We undertook a qualitative study with semi-structured interviews of 18 clinician managers (who have medical practitioner backgrounds) with formal leadership administrative roles. Interview transcripts were analysed with systematic text condensation.

**Results:**

All eligible participants approached in this context, agreed to participate and over 80% of the participants were male. We identified five themes: ‘Motivations for leadership’, ‘Pathways to managerial role’, ‘Challenges faced in management roles’, ‘Credibility through clinical practice’ and ‘Management skill cultivation and support’. Clinician managers progressed from being doctors to leadership roles through being encouraged to take on roles, while others felt pressure to take on leadership roles even if this was not a personal goal. Clinician managers described challenges such as feeling under-prepared, maintaining respect form colleagues through still participating in a clinical load, along with juggling priorities such as administrative tasks, managing budgets and performance managing other doctors.

**Conclusions:**

There needs to be an intentional and more structured approach to training and supporting clinician managers that considers the complex challenges faced by individuals (especially women) as they progress into these roles in the Australian tertiary health services context. There is a need to consider ways of supporting clinician managers to focus on management skills, effective mentorship and address perceptions around losing respect from colleagues if clinician managers cease their clinical loads. Further research is needed among the female medical workforce, along with research to understand if maintaining clinical loads when undertaking a clinical management role in fact leads to better effectiveness in contributing to better patient safety and quality outcomes. Such evidence may assist in addressing these social pressures among clinician managers, and contribute to addressing gender inequality among the clinical management workforce.

**Supplementary Information:**

The online version contains supplementary material available at 10.1186/s12913-021-06356-w.

## Background

Evolving with the rise of the New Public Management model across hospitals in the 1980s, there has been increasing demand for accountability from health services management teams worldwide [1–4]. Health services are now required to abide by strict accreditation standards in quality of care, patient safety and in the efficiency of care provision [1–7]. In Australia, the advent of activity-based funding have furthered emphasized the need for hospitals to be accountable for their clinical performance [8]. Activity-based funding is a system by which Australian state governments monitor, manage and administer the funding provided to public hospitals based on their provision of healthcare [8]. As a result, there has been an increased focus on encouraging and recruiting doctors to take on management positions and bridge gaps between the medical workforce and non-clinical administrators [9]. Internationally, over the past few decades, there has also been increasing focus on initiatives to increase the capacity of doctors to take on clinical management positions, including Europe [10], New Zealand [11] and the United States [12].

Clinical performance in hospitals is largely led by the medical workforce, with evidence to show that clinician managers contribute to improved clinical governance, performance and patient safety and quality within health services [13]. However from the management point of view, the medical profession is regarded to be difficult to engage in organizational and governmental endeavours [5, 14]. In particular, hospital management operated by non-medical executives have found it historically challenging to engage doctors within their organizations [2]. The lack of engagement with doctors have made it difficult for hospital management to address the increasing need for hospitals to be more efficient and to improve the safety and quality aspects of patient care, as these parameters are becoming increasingly scrutinized as part of the organization’s performance [2, 6]. Given the hierarchical nature of the medical profession, doctors are more inclined to listen to senior doctors with credibility, as opposed to listening to non-medical managers [2].

Higher agreement and collaboration between the medical workforce and non-clinical administrators has been shown to lead to an improved culture around patient safety and higher patient satisfaction with healthcare received at the hospital [7, 13]. This highlights the importance of encouraging doctors to undertake development into clinician manager roles, where they work more closely with health service administrators [15]. Due to the “highly socialized structure” of the medical profession, clinician managers were identified to be the conduit between hospital management and doctors within each organization [3].

When implemented properly, the role of clinician managers can lead to increased participation and involvement of medical staff in an organization’s strategic decision-making [2]. The benefits include enhanced patient safety and provision of clinical care, and enhanced patient advocacy. There would also likely be better professional relationships and communication between doctors and hospital management [3]. While another study by Norway found that there were three phases in clinician’s journey into management, along with a high level of frustration for being ‘thrown into leadership positions’ without adequate preparation [1, 5]. Little is known about the experiences of clinician managers in the Australian tertiary health service context.

This is the first Australian study conducted a tertiary hospital context that sought to:

1. Identify factors influencing doctors to become clinician managers in the Australian healthcare setting.
2. Understand the pathways and challenges faced by these doctors in becoming clinician managers

### Definition

For the purpose of this study, the term ‘clinician manager’ is used interchangeably with ‘medical manager’. In our study, a clinician manager is someone with a medical background (MBBS/MD) and who either works solely in an administrative/managerial capacity or is someone who has both managerial/administrative/education and clinical (patient management) roles.

## Methods

A qualitative approach with semi-structured interview of participants, was undertaken to explore the aims of the study by associate investigator Karen Rog who has experience and training in qualitative research methods. The interviewer, was assigned to undertake the interviews by the lead author due to having no pre-existing bias/relationships or conflicts of interest with the Clinician managers. Ethics approval for this study was obtained from the Western Health Low Risk Human Research Ethics Committee on 14 August 2016 (ref: HREC/16/WH/116). Written and informed consent was received from all participants. The interview guide and analysis were developed from the social constructionist theory perspective and with consideration of sociological theories that have been applied to the medical profession. Applying the sociologic theories of profession to the medical profession, doctors seek to attain influential positions e.g. to become a clinician manager, as a way to protect their profession from outside influences of non-medical managers [5]. In these theories, medical managers act as a buffer between hospital management and doctors so the medical profession within the unit/department continue to self-govern and maintain their autonomy [5].

### Recruitment

This study employed a convenience sampling approach to recruiting participants and was based in tertiary metropolitan health service in Victoria, Australia. To be eligible, participants had to be a qualified medical doctor (MBBS/MD) and be working in management positions within the health service. Eighteen doctors with management positions from medical and surgical departments across the health service were approached (via email) with a request to be interviewed for a qualitative research project. The participants identified were each emailed an invitation to participate in the research study and a Participant Information Statement document was attached to the email. All of the 18 doctors who were approached agreed to participate in the study and 15 of the participants were male.

### Participants

The participants selected were all medical practitioners. They have been each been appointed as Head of Units, Head of Department, Clinical Services Director positions, or Executive Director Medical Services.. Two participants were infrequently managing patients outside of their contracted roles with the health service. Within the health service, the two participants undertook non-clinician managerial and administrative leadership roles. Characteristics of the participants are presented in Table 1.

**Table 1:** Characteristics of participants (*N* = 18)

Characteristics	Number
**Gender**	
Female	3
Male	15
**Management level**	
Department/Unit	13
Divisional (consisting of several units)	4
Executive	1
**Clinical roles (management of patients)**	
No current clinical role	2
v30 to 80% of time	16
**Clinical expertise**	
Perioperative (Anaesthetics)	2
Medical	12
Surgical (Inclusive of Obstetrics & Gynaecology)	4

### Data collection procedure

A semi-structured interview guide was developed on the basis of existing literature (see supplementary information). The associate investigator conducted audio-recorded, face to face in-depth interviews with the 18 recruited participants, at the health service. Notes were also taken during the interviews. The participants, among other aspects of being a clinician manager, were interviewed about their career paths towards their current management positions, and their experiences of being a clinician manager. The semi-structured format allowed for participants to elaborate on specific points and discuss, in detail, their perspectives with the researcher. This allowed for themes to emerge which were not preconceived by the researchers involved. Each interview lasted between 45 to 60 min. Interviews were conducted until data saturation was reached, whereby no new themes arose were arising from the data, along with a high level of repetition in responses among participants [16]. In this study, only the associate investigator was aware of the identities of each of the research participants being interviewed. The associate investigator deleted any identifying information from the audio recordings. The de-identified audio-recorded interviews were then transcribed and the data analysed.

### Data analysis

The interview transcripts were transcribed and analyzed by systemic text condensation as per the widely accepted phenomenological analysis procedure. The steps of analysis include: (a) Reading all of the material to form an overall impression; (b) identifying units of meaning and subsequently coding for these units; (c) condensing and summarizing the contents of each coded group; and (d) generalizing the description and contents [17]. The transcribed interviews were analyzed by independently, by two of the research investigators (KR and DI, followed by LA) and the final themes generated were agreed upon by both investigators.

## Results

Five themes major themes were evident in the perspectives of the clinician managers, when describing the path to becoming a clinician manager and challenges experienced once in the role. Themes included ‘Motivations for leadership’, ‘Pathways to managerial role’, ‘Challenges faced in management roles’, ‘Credibility through clinical practice’ and ‘Management skill cultivation and support’.

### Motivations for leadership

Participants described a variety of motivations for pursuing clinical leadership roles. One motivation was the desire to lead and be proactive. These participants realised the only way to improve the current system was to take on a leadership role and have a voice in decision making.

“*… my desire was to be able to influence, to implement new ways of doing things and contribute …*” [Participant 13].

Another participant highlighted the important role of doctors in getting involved and leading change to enable better quality care.

*“…*. I *think that sometimes doctors don’t realize that to change things they are the ones who need to change things … … .there is no point complaining about things unless you are prepared to come on board and change things … .. It is up to the individual to step forward and change things …*.” [Participant 8].

A smaller proportion of participants recounted an innate and natural tendency to take on leadership roles. These participants had previous experiences of volunteering in quality improvement projects and had often participated in their health services’ clinical committees or their medical colleges’ working groups.

*“… .the people who seem to be very good leaders in my career seems to have been born that way ….”* [Participant 8].

Another participant confirmed their natural tendency towards leadership roles.

*“… .from a leadership point of view, I have always been involved with my college, local medical advisory committee etc … ..always drifted to those sorts of things ‘cause I do not mind doing it …”* [Participant 17].

Some participants viewed leadership roles as adjunct and necessary for survival and progression.

*“… ..the managerial stuff, I know it’s a necessary evil, to stay afloat in this day and age … .to be able to interface well with more senior managers ….”* [Participant 8].

Another felt management type roles were an evolution of a senior clinical role.

*“… .as you become more senior, the opportunities for career advancement are often by going into leadership/management roles … ..so it was not necessarily a planned move, and it was an evolution because I was becoming more senior within the organization ….”* [Participant 2].

### Pathways to a managerial role

The pathways to clinical leadership roles varied widely between participants. A cohort of participants who were already more proactive in pursuing leadership roles stressed the importance of taking career opportunities as they arise.

*“… the one thing I think I did right that has got me to a position where I am was taking opportunities. Take opportunities when they come and do not be afraid to take opportunities ….”* [Participant 8].

One participant described how it was important not to wait to be asked to apply and to go for opportunities regardless.

*“… opportunities arise …. I do not think you can expect to be tapped on the shoulder. Sometimes you have to put yourself out there and make it known you’re interested.* [Participant 14]

For some clinicians, their leadership abilities were noticed by colleagues and they were sought after by employment agencies. These clinicians were encouraged to pursue clinical leadership roles.

*“… ..people were telling me ‘you are actually good, you should think about going elsewhere and set up a unit’ and that gave me the self-belief that maybe I could ….”* [Participant 14]

Conversely, instead of chasing opportunities, some clinicians felt, due to circumstances beyond their control, they did not have a choice in accepting clinical leadership roles. These clinicians reported they never planned to become clinician managers. In these scenarios, it was difficult to find someone to take on the clinician manager role and it ultimately fell onto them to accept the position. However, once the clinician took on the leadership role, it was difficult to relinquish the position to a willing colleague. In these settings, the leadership role would evolve, and the clinician would gradually and inadvertently attain more credibility as a clinician leader.

*“… .because no one else would do it … (was) handed over the rights to me ….”* [Participant 16]

*“…. I never sat out for a leadership role specifically. As the opportunities came, I took them on and it evolved from there. Initially it was about ‘can you help us fix this or that?’ or ‘can you help us oversee this sort of thing?’ and then it gradually got to a point where I was approached to take on a deputy head of unit role and it evolved from there …”* [Participant 11]

### Challenges faced in management roles

Most participants reported a high administrative workload in their clinician manager roles.

As clinician managers, participants reported engaging in roster management, recruitment of staff, review of adverse clinical incidents, formulating and updating policies & guidelines, and regular engagement with hospital (clinical and non-clinical) management staff.

Despite the high workload, most participants reported receiving inadequate administrative support from their organization. Fourteen out of 18 clinician managers interviewed did not have administrative assistants and they found juggling administrative and clinical workloads to be challenging. One senior participant reported the long-standing lack of administrative support from hospital administration reflected the lack of understanding and appreciation of clinician manager roles. In the participant’s opinion, which makes the position of clinician managers significantly less enticing to prospective candidates.

“*Getting administrative support is really important … .the job is almost impossible because there are just so many things that need doing. The other challenge is the amount of emails which just bulk you down and are often not very productive”* [Participant 15]

Another highlighted how the administrative load can be highly burdensome.

“… *you find yourself pulled in all different directions and not being able to focus on anything for a reasonable period of time …”* [Participant 11]

One of the biggest challenges faced by clinician managers is managing fellow doctors. The clinician manager is able to relate to his/her fellow colleagues as a doctor. However, the clinician manager is also seen as a voice-piece for the organization. The clinician manager has to wrestle both the interests of his/her clinical craft group and the overall interests of the organization, from which frontline clinicians are often detached from.

Many participants acknowledged that clinicians, particularly senior doctors, are resistant to change. Senior doctors often work autonomously and are loathed to being instructed by anyone, let alone by ‘hospital management’ who they feel do not appreciate their work.

*“… whenever you try to change anything, there is always resistance to change. I think doctors are very resistant (to change) and their immediate reaction is always to say no”* [Participant 8]

Clinician managers understand their roles as conduits between front-line clinicians and hospital management and are thus often able to assuage the tensions or lack of understanding between both parties.

However, being the ‘middlemen’ poses a conflict for most participants. All participants identified themselves as clinicians first, and as managers second. Participants understand the viewpoints of fellow clinicians in their reluctance to adopt change or to conform with hospital administration’s instructions. However, in their roles of clinician managers, participants recognize that they themselves are part of the hospital administration team.

*“… I took a long time to understand the administrative parts of medicine (such as HR, rules & regulations, budgets, funding arrangements) and it would have helped if I had known all that from the beginning as it would have helped in the changes I was trying to get done without so many hiccups …”* [Participant 9]

A responsibility of clinician managers includes the propagation and dissemination of the organization’s vision and core values amongst fellow clinicians. This can be particularly challenging for clinician managers as fellow clinicians may feel they do not have a vested interest in the progress of the organization. Therefore, the clinician manager has to be empathetic to his/her fellow clinicians. However, the clinician manager risk being seen as being disconnected from his/her fellow clinicians if an organization’s agenda or by-laws are seen to be enforced through the manager.

*“… many clinicians do not have experience managing difficult staff and end up getting into trouble one way or the other … … there is a tendency for doctors to let some people get away with perhaps more than others, especially if someone is more senior or academical ….”* [Participant 8]

Most participants felt ill-equipped when they commenced clinician manager roles. In the participants’ opinion, many clinician managers had steep learning curves and struggled with the non-clinical aspects of management.

“… staff rosters, budgets … .we never get taught those in medical school or in specialty training …” [Participant 15]

### Credibility through clinical practice

All but one participant felt that it is inconceivable for clinician managers to cease managing patients. These participants were adamant that a clinician manager, no matter how technically skilled or experienced, risk losing credibility amongst his/her fellow clinicians if he/she undertake an entirely administrative role.

*“If you got people who just wanted to manage, I don’t think they’d have the respect of the unit. You’ve got to be at the call-face at least part of the time to show that you understand what the demands are, so if you’re there you can see with your own eyes and you lead by example”* [Participant 19]

Another participants also summarised the link between credibility as a manger and clinical practice.

*“… I could see people around me that were losing clinical credibility as leaders because they were not doing enough clinical work. I do not think you can get other consultants to be doing things unless you understand what their problems are …”* [Participant 9]

Most (17 out of 18) participants thoroughly enjoyed managing patients and felt it was their calling and life-long vocation. Some participants reported enjoying the clinical aspects of their position significantly more than the administrative component, which poses an ongoing challenge for them.

“*It’s very important because that’s the reason I became a doctor. Ultimately it’s to care for patients”* [Participant 11]

Conversely, some participants were attracted to the administration aspect of being a clinician manager precisely because it is challenging and stimulating, unlike managing patients which came easily for them.

### Management skill cultivation and support

All of the participants cited the importance of under-going additional training in response to feeling ill-equipped when commencing clinical leadership roles. All participants either enrolled in formal medical management courses, pursued organizational leadership courses or sought informal self-directed learning opportunities. Some of the participants had attained a post-graduate qualification in healthcare management, medical education or in public health.

*“… if you have interest in that field, then you should go off and do those as part of a Masters of Public Health or in a Medical Management qualification. It gives you a much deeper insight than you ever get during medical school or specialist training …”* [Participant 11]

Another also described a need for additional education in their management role.

*“… I was unhappy with how we were assessing trainees in the workplace … So I went and did a graduate certificate in education …”* [Participant 8]

All participants benefited from having a mentor or mentors when they commenced their clinical leadership roles. In all cases, the process of mentorship took place informally and, in some instances, the mentor had been the person who encouraged the participant to pursue the leadership position. The mentors described were clinician managers themselves and, in most cases, were the predecessors of the current participants in their leadership roles.

*“… my two direct bosses had been great … the job would have been impossible without their support and experience …”* [Participant 8]

## Discussion

Our study found that multiple factors influenced Australian doctors to become clinician managers including wanting to contribute to positive change within the health service, being encouraged by others to apply for leadership roles, or feeling pressure to take on a clinical leadership role. Once in such roles, the doctors experienced multiple challenges including a high administrative burden, as well as difficulties in managing other doctors. The participants also emphasized the importance of maintaining a clinical load to ensure they preserved respect from their fellow clinical colleagues, as full-time non-clinical administrators were not as well respected in their roles. It is important to note, that in this Australian tertiary health service context, 83% of the participants were male, reflective of the demographic composition of management in this particular setting, at the time of the study. Another recent study, that conducted a qualitative enquiry in to the experiences of 15 clinician managers in Hong Kong [18], also included a male dominated sample (*n* = 1 female), noting this as one of the study limitations, and was representative of the gender mix in management in the Hong Kong tertiary setting. A gender imbalance between men and women in the medical leadership roles, despite increasing numbers of graduating female doctors, has been an issue for decades, not only in Australia but worldwide [19, 20]. The experience of female doctors in similar contexts requires further investigation. Most urgently, research that seeks to identify barriers and facilitators for female doctors in seeking to progress to clinical management roles is needed. The mostly male participants in this study described different pathways to managerial roles, with some participants feeling ‘forced’ into roles they did not feel prepared for, while others actively sought career progression to management. Exploration of how gender bias implicates pathways to management for female doctors requires particular attention in order to progress gender equality in clinical management in this context.

Generally participants felt ill-equipped to be in management roles and all of the participants cited the importance of under-going additional training in response to feeling ill-equipped when commencing clinical leadership roles. Most of the participants reported a high administrative workload in their clinician manager roles and inadequate support which reflected the lack of understanding and appreciation of clinician manager roles. One of the biggest challenges faced by clinician managers is managing fellow doctors, as they understood both the doctor’s needs, but had to juggle organisational pressures. This is consistent with previous literature from the international context, that doctors often progress to clinical management roles, and find themselves unprepared, unskilled and unsupported, and exists in the Australian tertiary setting [5, 6, 14].

All but one participant felt that it is inconceivable for clinician managers to cease managing patients. These participants were adamant that a clinician manager, no matter how technically skilled or experienced, risked losing credibility amongst their fellow clinicians if they were to undertake an entirely administrative role. Although participants expressed the importance of maintaining a clinical load alongside their management role (or being a ‘hybrid manager’), other studies have found that doctors who are ‘hybrids’ in their roles may be less likely to be effective at their non-clinical management role [2, 21]. Our findings reflect the wider literature that there is an existing perception among clinicians, that clinical work is viewed as more important than management. Social pressure among the medical workforce, does not necessarily translate into these leaders are more effective in their roles [2] and appears to be more important in informing perceptions that the clinician manager understands the experience of doctors (irrespective of previous clinical experience). A qualitative study of clinician managers in the US also found that the participants placed high value on their identity as a clinician and felt this made them better leaders as their careers progressed [21]. They also expressed, that upholding their clinical knowledge, gained respect of their medical colleagues in superiority to their capacity in management or skills as an organizational leader [21]. Similarly, Spehar et al., 2012, also described having a medical background alone does not sustain a clinician manager’s credibility among his/her medical peers. The study found that participants perceived a doctor’s ongoing clinical practice, to be deemed essential for the maintenance of a medical manager’s credibility [5]. More studies are needed to understand associations between those who maintain their clinical load, alongside managerial roles, and the effectiveness of these leaders in contributing to better outcomes from both a patient and organizational perspective. Such evidence could be used to generate change in the perceptions of doctors around social pressures to juggle both clinical and managerial tasks, particularly if this does not influence leadership effectiveness. It may also contribute to relieving the pressure on doctors who find continuing both clinical and management roles to be a challenge, and instead focus on developing their managerial and leadership skills and performance. Future research should also consider targeted recruitment of clinician managers who do not have clinical responsibilities, to understand perceptions around the importance of maintaining a clinical load in those who have not continued to. This research could also extend to international settings, to understand if there are similar pressures faced by clinical managers across the globe.

Most of the participants in this study described feeling unskilled to undertake management roles, and when taking the step into the role, felt overwhelmed and under-supported. This is consistent with other literature form around the world that describes the same experiences of doctors in clinical management roles and poses a risk to the sustainability of the clinical management profession [5, 14, 21, 22], and evidentially exists for Australian doctors in this setting. Globally, there is increasing recognition for more formalized and structured approaches to leadership and administrative training for clinician managers [23]. Until recently, training for clinician managers has been uncoordinated and variable in standards. In recognition of the lack of leadership and management training in the undergraduate medical school curriculum, many jurisdictions have introduced formalized management training as a competency for medical and surgical specialty training programs [24, 25]. For example, the specialty training colleges in Canada and Australia integrated the CanMEDS framework into their curriculum [23–25]. In Australia for example, the Royal Australasian College of Medical Administrators (RACMA) is one of several medical colleges accredited by the country’s medical board to provide a specialty-specific training program [24]. Such training programs need to consider the unique challenges clinician managers face, along with social pressures such as maintaining a clinical load, to ensure perceived respect is maintained with colleagues, not just in Australia, but around the world. Future research into how clinician managers can be better supported is essential into ensuring long term sustainability of these roles, along with ensuring that clinician managers have the skills and support to undertake these roles to an exceptional standard. Global comparisons between the experiences of clinical managers may also reveal similar challenges which may be better addressed with a more unified and consistent approach.

### Strengths and limitations

This study used a purposive sampling approach to identify key people working within the tertiary health service system and understand their experience in progressing to a clinician manager role, along with the challenges they have experienced in the roles. A significant strength of this study is that the recruitment process resulted in a very informed sample and a second strength is that all key informants approached agreed to participate. A potential limitation of this study is that the sampling approach relied on the potential key informant being accessible via email or known to be in a clinical management role, which may have potentially excluded participants who were new to such roles and not identified by the researchers. A further limitation was that, although reflective of the management groups demographic composition at the time of study, women were largely underrepresented. Barriers to women entering leadership roles in this context needs further investigation to reduce gender inequality in clinical management roles. In addition to this limitation, we did not explore whether the perspectives of participants differ by ethnicity or cultural background, along with other indicators of diversity, which should also be considered in future research.

## Conclusion

The clinician manager is key figure to bridging the ethos propagated by a health services’ management team and the medical staff. There needs to be an intentional and more structured approach to training and importantly, supporting clinician managers that considers the complex challenges faced by individuals as they progress into these roles. In this context, there is a need to consider ways of supporting clinician managers to focus on management skills, seek mentorship and address perceptions around losing respect from colleagues if clinician managers cease their clinical loads, to focus their energy on management. Further research is needed to understand the experience of women and if maintaining clinical loads when undertaking a clinical management role leads to better effectiveness of the leader in contributing to better patient safety and quality outcomes. This may assist in addressing social expectations among the medical workforce within this tertiary healthcare setting.

## Supplementary Information

**Additional file 1.**

## Data Availability

The raw data is not available for this study due to ethics approval guidelines and confidentiality.
