# Supplementary figures and images for: The challenges of becoming and being a clinician manager: a qualitative exploration of the perception of medical doctors in senior leadership roles at a large Australian health service
Source: BMC Health Serv Res. 2021 Apr 15;21:351. doi: 10.1186/s12913-021-06356-w (PMC8051065; doi:10.1186/s12913-021-06356-w)

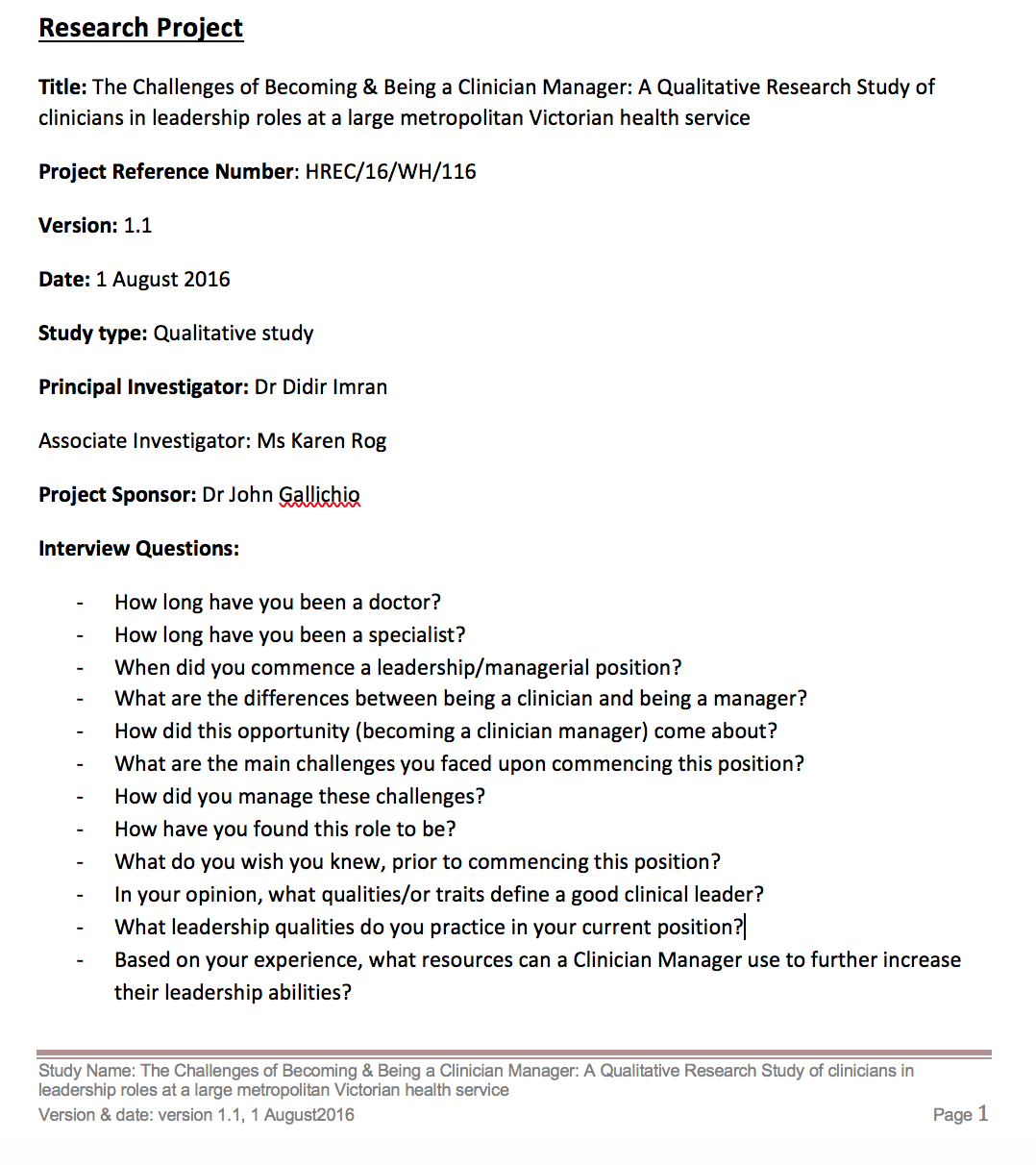

Supplement: Supplementary file 1 — Additional file 1. [file 12913_2021_6356_MOESM1_ESM.docx]
